#  Acquired Angioedema Related to Transient C1- Inhibitor Deficiency Triggered by *Mycoplasma pneumoniae* Infection: A Case Report

**DOI:** 10.3390/reports8030164

**Published:** 2025-09-01

**Authors:** Athanasia-Marina Peristeri, Olympia Akritidou, Anna Nikopoulou, Konstantina Theodoridou, Michail Leontakianakos, Christina Chrysanthi Theocharidou, Georgios Pilianidis

**Affiliations:** 1 Department of Internal Medicine, G. Papanikolaou General Hospital of Thessaloniki, 57010 Thessaloniki, Greece; ak.olympia@gmail.com (O.A.); nikopanna@gmail.com (A.N.); teokonstantina@gmail.com (K.T.); gpilianidis@yahoo.gr (G.P.); 2 Pulmonary Department, Aristotle University of Thessaloniki, G. Papanikolaou General Hospital of Thessaloniki, 57010 Thessaloniki, Greece; leontasmike@gmail.com; 3 1st Intensive Care Unit, G. Papanikolaou General Hospital of Thessaloniki, 57010 Thessaloniki, Greece; chrtheoch@gmail.com

**Keywords:** *Mycoplasma pneumoniae*, acquired angioedema, C1-inhibitor deficiency, cold agglutinins

## Abstract

**Background and Clinical Significance**: Acquired angioedema (AAE) is a rare and potentially life-threatening condition characterized by acquired deficiency of C1-inhibitor (C1-INH) resulting in hyperactivation of the classical complement pathway. AAE occurs in association with malignancies or autoimmune diseases. Infectious triggers are rarely encountered, and the underlying mechanisms have yet to be completely clarified. **Case Presentation**: This case involves a previously healthy 19-year-old male who was admitted with *Mycoplasma pneumonia* and oral ulcers, subsequently developing unilateral facial angioedema. Laboratory studies demonstrated reduced C4, decreased levels and activity of C1-INH, and reduced C1q, all consistent with acquired C1-INH deficiency. These findings were attributed to the presence of cold agglutinins, which are frequently observed in *Mycoplasma pneumoniae* infections. Following treatment with icatibant, a bradykinin B2 receptor antagonist, the patient’s angioedema resolved rapidly. An exhaustive workup found no evidence of underlying systemic disorders, and the patient did not experience any angioedema attacks following resolution of the infection. **Conclusions**: The presence of cold agglutinins, commonly associated with *Mycoplasma* infections, can precipitate a decline in C1-INH levels, resulting in complement pathway dysregulation. This disruption leads to an excess of bradykinin, followed by increased vascular permeability and localized edema.

## 1. Introduction and Clinical Significance

Angioedema (AE), a non-pitting edema of the dermis and subcutaneous tissues, is traditionally classified into three main categories based on underlying mechanisms: histamine-mediated angioedema, bradykinin-mediated angioedema, and angioedema of unknown origin. Differentiating between these types in acute settings is often challenging due to a lack of timely laboratory results [1,2].

Acquired angioedema due to C1-inhibitor deficiency (AAE-C1-INH) is a particularly uncommon disorder characterized by autoantibody-mediated consumption of C1-INH, leading to dysregulation of the classical complement pathway and unchecked bradykinin generation [3]. In the clinical setting, AAE-C1-INH typically affects adults and is often associated with underlying lymphoproliferative disorders, monoclonal gammopathy of undetermined significance, or autoimmune conditions. Rare associations with infections have been reported, although these remain poorly understood [4]. In a study published by an international consensus group, it was reported that most patients with AAE-C1-INH demonstrate low C4 and C1q levels, as well as diminished C1-INH antigenic concentration and function [5].

While *Mycoplasma pneumoniae* infections cause a variety of well-documented extrapulmonary manifestations, the development of angioedema in association with this pathogen remains uncommon [6,7]. Furthermore, the potential for infectious agents to trigger acquired angioedema through indirect mechanisms has not been adequately established in clinical practice [8].

This report presents a unique case of AEE-C1-INH in a previously healthy 19-year-old male occurring concurrently with the presence of cold agglutinins in a setting of *Mycoplasma Pneumoniae* infection, suggesting a potential infectious trigger for this rare condition.

## 2. Case Presentation

A 19-year-old Caucasian male with no documented pre-existing medical conditions presented to the emergency department (ED) with a one-week history of fever and oral ulcers. His symptoms began with a nonproductive cough, for which he was initially evaluated at a rural health center. A chest X-ray (CXR) performed at that time showed hypoventilation of the right lower lung field with early infiltrates, and he was started on amoxicillin/clavulanic acid. Over the following days, he developed daily fevers up to 39 °C, along with progressive swelling and ulcerative lesions of the lips and oral cavity.

Due to persistent and worsening symptoms, the patient was referred to our tertiary center for further evaluation. Upon arrival, he was assessed by otorhinolaryngology and by oral and maxillofacial surgery teams, and hydrocortisone and antihistamines were administered, but without significant improvement. He was subsequently admitted to the internal medicine department.

On clinical examination the patient presented unilateral rales. Peripheral oxygen saturation on room air was 94%. Based on these findings a diagnosis of community-acquired pneumonia was considered the most probable. The patient was promptly initiated on empirical antibiotic therapy with levofloxacin. Blood and urine cultures were obtained, and sputum samples were sent for routine bacterial culture; a respiratory PCR panel test was also conducted, which returned positive for *Mycoplasma pneumoniae*. Regarding the oral lesions, the differential diagnosis included localized Stevens–Johnson syndrome of the oral mucosa related to amoxicillin/clavulanic acid administration or the underlying infection, as well as ulcers associated with the presence of cold agglutinins (which are frequently present in *Mycoplasma* infections). In light of these findings, an oral biopsy was performed, with results showing non-specific inflammatory changes. Nevertheless, the patient presented a favorable clinical response to topical dexamethasone.

Due to a mild respiratory compromise, an urgent high-resolution computed tomography (HRCT) of the chest was performed, which revealed ground-glass infiltrates in the posterior basal segment of the lower right lobe, as well as consolidation in the posterior basal segment of the right lower lobe, consistent with pneumonia. Due to a mismatch between hematocrit and hemoglobin values, which resolved after an incubation of the sample at 37 °C, the patient was evaluated and tested positive for the presence of cold agglutinins. Additional findings included a positive direct Coombs test (+2), and low C4 levels with normal C3 levels. In spite of these results, the patient presented no clinical signs of hemolysis.

**Figure 1:**
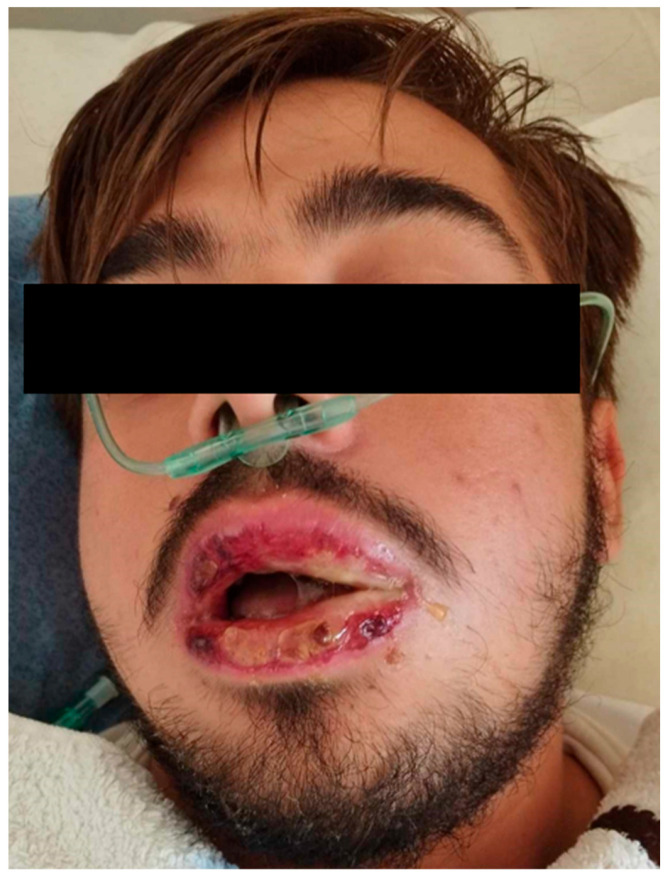
Angioedema and ulcerative lesions of the lips and oral mucosa. Patient consent was obtained.

During the third day of hospitalization, the patient abruptly developed edema of the left side of the face without urticaria (Figure 1). There were no complaints of dyspnea or pruritus, and no associated tongue or uvula edema was observed. This presentation, alongside the low C4 levels and the lack of clinical response to the administration of corticosteroids and antihistamines, raised strong clinical suspicion of acquired angioedema [5]. Treatment with a single dose of icatibant, a selective bradykinin B2 receptor antagonist, resulted in complete resolution of the facial swelling within a few hours [9]. Further laboratory investigations revealed decreased C1-INH antigen level and function, as well as a decreased level of C1q.

Following the third day of hospitalization, the patient showed significant clinical improvement with fever resolution. He was subsequently gradually weaned from oxygen supplementation. Laboratory monitoring showed a gradual declining rate of inflammatory markers and the patient was eventually discharged.

A month later, the patient was re-evaluated and repeat testing revealed no presence of cold agglutinins. Normal complement studies (C4, C1-INH antigen/C1 INH function) were also carried out. Laboratory results obtained from the patient during admission and follow-up are presented in Table 1. Because the majority of patients presenting with AAE are found to have an underlying malignancy or autoimmune disorder, additional imaging and laboratory investigations were performed. A chest and abdomen CT scan produced unremarkable findings, and further clinical and laboratory evaluation did not demonstrate the presence of an autoimmune condition [9]. At this stage, taking into consideration the patient’s negative family history of AE, and the lack of recurrent AE attacks, as well as the extremely low levels of C1q, the diagnosis of AAE-C1-INH was established. According to Cicardi et al., after confirming C1-INH deficiency by detecting low C4 and a reduced C1-INH antigen protein/functional level, measuring C1q levels is key in differentiating between AAE-C1-INH and hereditary angioedema (HAE). It is important to note that C1q levels are decreased in 70% of patients with AAE-C1-INH but are not affected in HAE [3].

## 3. Discussion

*Mycoplasma pneumoniae* infections have been associated with numerous extrapulmonary manifestations, including acute disseminated encephalomyelitis, Guillain–Barré syndrome, and myocarditis [6]. Dermatologic conditions, such as erythema nodosum and erythema multiforme have also been reported [10,11,12]. A review of the literature indicates that only two documented cases of *Mycoplasma pneumoniae*-associated angioedema have been described. Neither case appears to reference bradykinin-mediated angioedema. In the first case, a 40-year-old woman developed non-episodic angioedema with marked eosinophilia, urticaria, fever, weight gain, and elevated serum IgM several weeks after a generalized pruritic rash and confirmed *M. pneumoniae* infection. Her course was notable for persistent skin lesions, lymphadenopathy, and laboratory evidence of immune activation, with symptoms resolving under corticosteroid therapy and no relapse during follow-up. In this setting, AE was part of a clinical entity known as Gleich syndrome, which is classified in the broad category of idiopathic hypereosinophilic syndromes [13]. In the second case, a previously healthy 5-year-old boy with *M. pneumoniae* pneumonia presented with acute, massive AE of the lower lip in the absence of other mucocutaneous findings or drug-induced triggers. Laboratory workup revealed normal C4 and C1-INH levels and function, thus excluding AAE-C1-INH. This patient responded favorably to corticosteroid and antihistamine administration, suggesting AE as part of a histamine-mediated reaction, even in the absence of urticaria [7].

AE is classified into three main categories based on distinct pathophysiological mechanisms. Two overarching categories include mast-cell-mediated AE, also known as histaminergic AE, and bradykinin-mediated AE (BK-AE). However, in many cases of AE the underlying process remains unclear. In the worldwide academic literature, this subgroup is referred to as acquired AE of unknown origin [1]. In mast-cell-mediated AE, histamine is the principal mediator, leading to an immediate type I hypersensitivity reaction. The early recognition of this clinical entity is facilitated when other signs and symptoms, such as urticaria, flushing, and pruritus are present. In contrast, BK-AE constitutes a rare clinical entity, the pathogenesis of which is not yet fully understood. A promising pathophysiological mechanism includes interactions between bradykinin, high-molecular-weight kininogen, and kallikrein [14]. High clinical suspicion is of great importance regarding the early recognition of BK-AE, as delays in therapy may lead to fatal outcomes. The classic presentation includes non-pitting, nonpruritic, asymmetrical, localized edema of subcutaneous or submucosal tissues, which can help guide the differential diagnosis. There are two distinct groups of BK-AE: BK-AE with C1-INH deficiency, which is further divided into acquired (AAE-C1-INH) and hereditary (HAE); and BK-AE without C1-INH deficiency, which includes hereditary (mutations in the F12 or PLG genes), drug-induced, and non-mast-cell-mediated BK-AE [15].

AAE-C1-INH is a rare condition characterized by low levels of C1-INH, C1q, C4, and C2, alongside recurrent AE symptoms on the grounds of aberrant activation of the complement pathway [3]. Underlying conditions, such as lymphoproliferative diseases, monoclonal gammopathy of undetermined significance, autoimmune disorders, and solid organ tumors have been associated with the production of C1-INH neutralizing autoantibodies, leading to its consumption [2,4]. The resulting overproduction of the vasoactive mediator bradykinin leads to increased extravasation of intravascular fluid and formation of edema in the subcutis and/or the submucosa [16]. As with other types of BK-AE, AAE-C1-INH typically presents with recurrent cutaneous swelling in the absence of urticaria. In patients without a family history of AE, AEE-C1-INH should be considered in the differential diagnosis when these symptoms are present [5]. Given the lack of specific approved treatment options for AEE-C1-INH, treating the underlying condition takes precedence in preventing future attacks [2,3].

To our knowledge, C1-INH-AEE has rarely been associated with infectious etiologies [8,17,18]. Current evidence supports that patients with acquired AE should undergo a thorough clinical and laboratory evaluation in search of possible underlying malignant or autoimmune conditions. In most such instances, a paraprotein, cryoglobulin, or autoantibody was thought to activate C1, resulting in the depletion of C1-INH [2,3]. Cold agglutinins are autoantibodies that typically belong to the IgM class, and have the property of agglutinating red blood cells (RBCs) at temperatures below 37 °C. They are directed against red blood cell antigens, resulting in activation of the complement system. The IgM-RBC complexes, along with activated complement components, can deposit on the endothelium, the inner lining of blood vessels causing endothelial injury and end-organ damage [19]. They are frequently detected in the context of infections, such as *Mycoplasma pneumoniae*, as well as lymphoproliferative disorders [20,21]. As illustrated in our case, the presence of cold agglutinins is often associated with skin ulcers. Other findings include anemia-related symptoms due to RBC lysis, as well as circulation-related symptoms such as acrocyanosis and Raynaud’s phenomenon [21]. Thus, cold agglutinins may trigger the reduction in C1-INH levels, consequently leading to dysregulation of the complement pathway. In this context, infectious causes of cold agglutinin disease could serve as a potential precipitating event for the development of C1-INH-AEE [22,23].

## 4. Conclusions

This case report adds to the limited evidence that infectious causes, such as *Mycoplasma pneumoniae*, can act as precipitating factors for AAE-C1-INH, even in previously healthy patients without traditional risk factors. Serological evidence of cold agglutinins, often associated with infections, may play a crucial role in complement activation and C1-INH consumption, serving as a potential mechanism for AAE in these clinical settings. Timely detection and elimination of the underlying trigger are essential for optimal management and prevention of recurrent AE attacks, while the use of targeted agents such as bradykinin receptor antagonists is crucial for preventing life-threatening complications in acute episodes. While a detailed and thorough assessment for the presence of associated comorbidities remains essential in all cases of acquired angioedema, clinicians should also consider infectious triggers, particularly when initial investigations are unrevealing.

In our case, the patient’s C1q levels were markedly decreased during the acute episode, supporting the diagnosis of acquired angioedema, rather than hereditary. This finding, along with the patient’s lack of family history or previous episodes, helped confirm AAE as the final diagnosis. Nonetheless, it is important to note that rare exceptions exist where hereditary angioedema may present with low C1q [24].

## Figures and Tables

**Table 1:** Test values obtained from the patient during admission and follow-up.

Test	Reference Values (Adults)	Admission to Hospital	Follow-up
Hemoglobin (g/dL)	14.0–18.0	16.5	16.9
Eosinophils (×1000/mmc)	0.05–0.50	0.41	0.18
Thrombocytes (×1000/mmc)	150–450	274	258
ESR (mm/h)	<25	45	20
CRP (mg/dL)	<0.5	15.3	0.06
AST (U/L)	8–33	28	24
ALT (U/L)	4–36	22	20
GGT (U/L)	5–40	27	31
ALP (IU/L)	44–147	87	76
Total protein (g/dL)	6–8.3	7.1	7
Serum albumin (g/dL)	3.4–5.4	4.2	4.4
ß2-Microglobulin (mg/L)	1.0–1.8	1.1	1.3
IgG (mg/dL)	751–1560	1320	1370
IgA (mg/dL)	84.5–499	104	90.8
IgM (mg/dL)	46–304	303	276
IgE (IU/mL)	0–100	50	54
C1-INH (mg/dL)	22–34	18	31
C1-INH activity	>67%	45%	90%
C1q (mg/dL)	10–25	2	24
C3 (mg/dL)	79–152	151	101
C4 (mg/dL)	16–38	2.1	19.1
Cold agglutinin (dil.)	<1/32	1/128	Not detected
LDH (UI/L)	<250	302	237
Rheuma factor (IU/mL)	<20	<20	<20
ANA	Negative	Negative	Negative

## Data Availability

No new data were created or analyzed in this study. Data sharing is not applicable to this article.

## Abbreviations

The following abbreviations are used in this manuscript:

AAE	Acquired angioedema
C1-INH	C1-inhibitor
AE	Angioedema
AAE-C1-INH	Acquired angioedema due to C1-inhibitor deficiency
ED	Emergency department
CXR	Chest X-ray
HRCT	High-resolution computed tomography
BK-AE	Bradykinin-mediated angioedema
RBCs	Red blood cells
